# Severe fever with thrombocytopenia syndrome presenting as hemophagocytic syndrome: two case reports

**DOI:** 10.1186/s40064-016-2010-2

**Published:** 2016-03-22

**Authors:** Akihito Kitao, Ryuji Ieki, Hiroki Takatsu, Yuki Tachibana, Masaaki Nagae, Takuya Hino, Hitoshi Nakaji, Masayuki Shimojima, Masayuki Saijo, Masanobu Okayama, Tsuneaki Kenzaka

**Affiliations:** Department of General Medicine, Public Toyooka Hospital, 1094, Tobera, Toyooka, Hyogo 668-8501 Japan; Division of Medical Oncology/Hematology, Department of Medicine, Kobe University Graduate School of Medicine, Kobe, Japan; Department of Respiratory Disease, Public Toyooka Hospital, Toyooka, Japan; Department of Virology 1, National Institute of Infectious Disease, Tokyo, Japan; Division of Community Medicine and Medical Education, Kobe University Graduate School of Medicine, Kobe, Japan; Division of Community Medicine and Career Development, Kobe University Graduate School of Medicine, Kobe, Hyogo Japan

**Keywords:** Severe fever with thrombocytopenia syndrome, Ticks, Consciousness level, Hemophagocytic syndrome

## Abstract

**Introduction:**

Severe fever with thrombocytopenia syndrome (SFTS) is an emerging infectious disease that was first reported in China in 2011. However, it is now endemic in Japan, and the SFTS viruses in Japan and China have evolved independently. Its fatality rate is 26.5 % in Japan, and the viral load is related to morbidity.

**Case description:**

We encountered two patients with SFTS. Case 1 is a 72-year-old woman who visited our hospital owing to severe fatigue, diarrhea, and nausea. Her consciousness level score on the Glasgow Coma Scale was 14 points, and her serum lactate dehydrogenase level was 646 IU/L. Case 2 is an 82-year-old woman who visited our hospital owing to diarrhea and general fatigue. Her consciousness level score on the Glasgow Coma Scale was 11 points, and her serum lactate dehydrogenase level was 935 IU/L.

**Discussion and evaluation:**

Both patients had hemophagocytic syndrome and presented with similar symptoms. Although both were treated with similar drug regimens, their clinical courses were different: after treatment, the 72-year-old woman survived whereas the 82-year-old woman died. In addition to age, the two patients differed in terms of time between symptom onset and treatment initiation, consciousness level, viral load, and extent of elevation of liver enzyme levels.

**Conclusions:**

The viral load, which is a predictor of morbidity, was associated with the level of consciousness and the serum lactate dehydrogenase level, both of which might be useful for predicting death in patients with SFTS.

## Background

Severe fever with thrombocytopenia syndrome (SFTS), an emerging infectious disease, was first reported in China in 2011 (Yu et al. 2011). The major clinical symptoms and findings of this disease include fever, emesis, diarrhea, lymph node swelling, neutropenia, and thrombocytopenia. The cause of the illness is infection with the SFTS virus, classified under family *Bunyaviridae*, genus *Phlebovirus*. The SFTS virus is transmitted by ticks, and hence, infection mainly occurs between early summer and late autumn (Zhang et al. 2012a). The fatality rate for SFTS in China was approximately 30 % when the disease was first reported, but has fallen to 12 % owing to increases in the number of reported cases and consequent advances in diagnostic procedures (Gai et al. 2012a). Supportive care remains the standard treatment.

In Japan, the first case of SFTS was reported in January 2013 (Takahashi et al. 2014); since then, 166 cases have been registered at the National Institute of Infectious Disease (Tokyo, Japan) as of December 2, 2015. The fatality rate in Japan is 26.5 % (of the reported 166 patients, 44 patients died), which is higher than that in China (12 %). Since 2012, all cases of SFTS have been reported from western Japan (Takahashi et al. 2014). The genome sequence of the SFTS virus identified in Japan is phylogenetically different from that identified in China. These facts demonstrate that SFTS is endemic in Japan and that the SFTS viruses in Japan and China evolved independently (Takahashi et al. 2014).

Although it was recently reported that type-I/II interferon (IFN) plus ribavirin drastically reduced SFTS virus titers in vitro (Shimojima et al. 2015), a standardized treatment for SFTS other than supportive care has not yet been established. According to previous reports, the viral load is related to morbidity (Yoshikawa et al. 2014; Sun et al. 2012; Gai et al. 2012b). Herein, we report two patients with SFTS who visited the Toyooka Public Hospital. Hemophagocytic syndrome was diagnosed in both patients according to the HLH2004 criteria (Henter et al. 2007). Although both had similar symptoms including alterations in consciousness levels and gastrointestinal manifestations and received similar drug regimens, their clinical courses were different. The viral load correlated with the level of consciousness and the serum level of lactate dehydrogenase (LDH). We discuss the prognosis of SFTS in these two cases.

## Case presentation

Written informed consent was obtained from the patients and/or their families for publication of this case report and the accompanying images.

### Case 1

A 72-year-old female farmer in a mountainous area was referred to the Public Toyooka Hospital by her family physician owing to fever (body temperature, approximately 38 °C), leukopenia, thrombocytopenia, and a mild consciousness disturbance. Four days previously, she had experienced severe fatigue, diarrhea, and nausea.

**Fig. 1:**
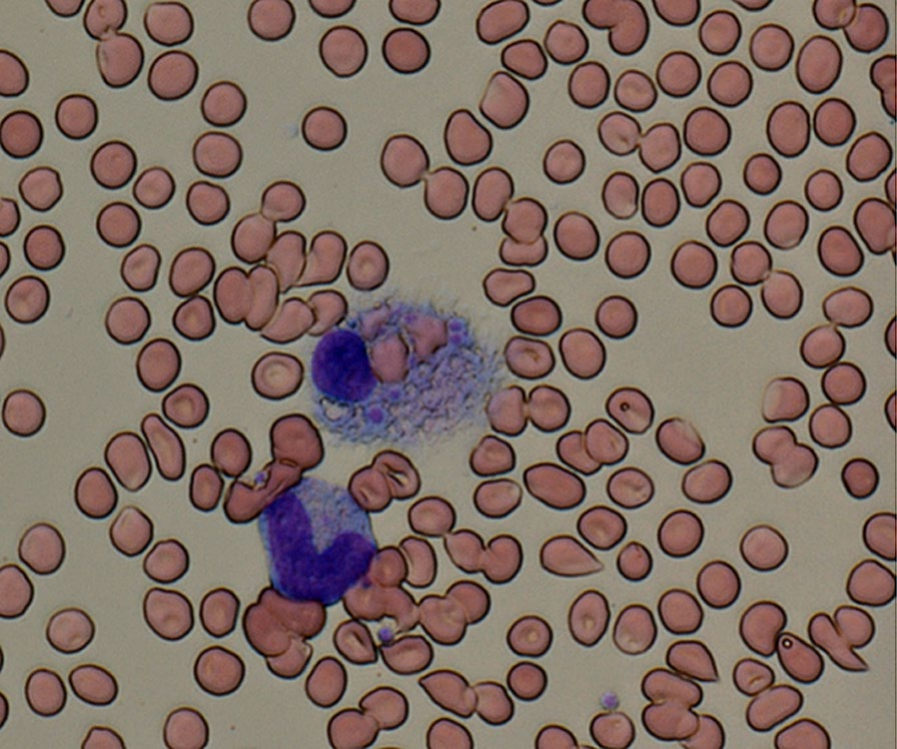
Bone marrow smear findings for case 1. Red blood cells are ingested by a macrophage (May-Giemsa staining, ×400 magnification)

The patient had been receiving amlodipine at 5 mg/day for 10 years, as prescribed by her family physician. Her score on the Glasgow Coma Scale (GCS) was 14 points (eye opening, 3; verbal response, 5; and motor response, 6), and her vital signs were as follows: temperature, 37.6 °C; pulse rate, 70 beats per minute (regular); blood pressure, 99/58 mmHg; and respiration rate, 18 breaths per minute. A physical examination revealed splenomegaly and swelling of the inguinal lymph nodes. Contrast-enhanced computed tomography revealed splenomegaly and swollen axillary and inguinal lymph nodes. Laboratory findings were as follows: white blood cell (WBC) count, 1400/µL (lymphocyte count, 80/µL); red blood cell (RBC) count, 478 × 10^4^/µL; hemoglobin (Hb) level, 13 g/dL; platelet count, 7.2 × 10^4^/µL; aspartate aminotransferase (AST) level, 117 IU/L; alanine aminotransferase (ALT) level, 33 IU/L; LDH level, 646 IU/L; creatinine phosphokinase (CPK) level, 595 IU/L; blood urea nitrogen (BUN) level, 26.4 mg/dL; serum creatinine (Cr) level, 0.89 mg/dL; fibrinogen level, 134.2 mg/dL; fasting triglyceride level, 256 mg/dL; soluble interleukin-2 receptor (sIL-2R) level, 1320 U/mL (normal, 149–519 U/mL); and ferritin level, 1908 ng/mL. A capillary blood smear test showed no abnormal cells. A bone marrow aspiration smear showed that RBCs were ingested by macrophages (Fig. 1). The patient met six of the eight HLH2004 diagnostic criteria and was therefore diagnosed with hemophagocytic syndrome and admitted to the hospital. Although a workup was performed to determine the cause of this syndrome, no previously described etiologic agents or diseases were detected (e.g., malignant lymphoma, Epstein-Barr virus, human immunodeficiency virus, systemic lupus erythematosus, adult-onset Still’s disease, and rheumatoid arthritis).

**Fig. 2:**
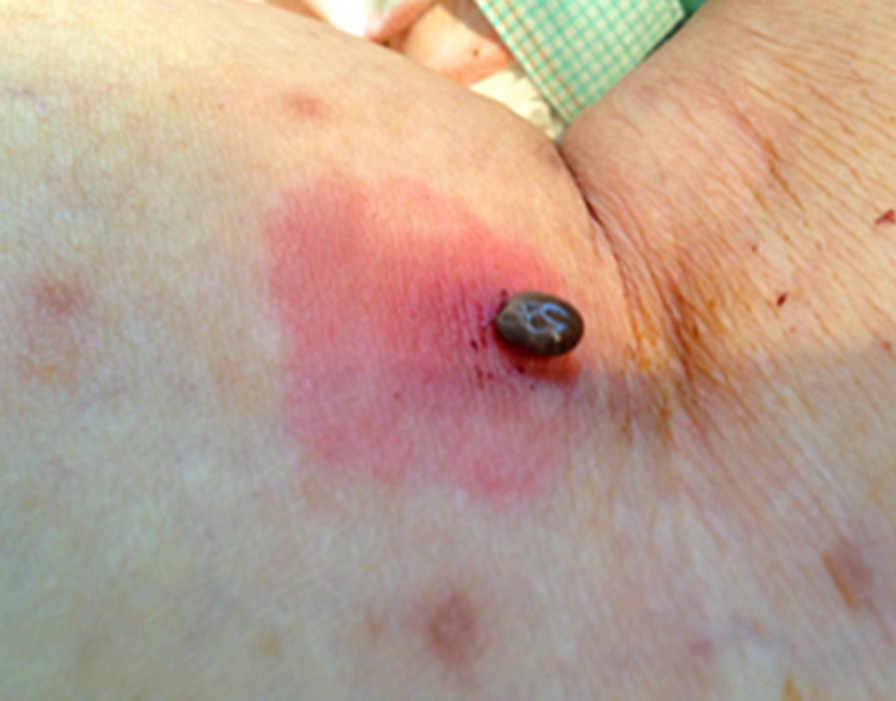
Tick biting the left popliteal fossa in case 1. The patient had *red* flares around the bite site

The following day, a tick was found attached to the skin of the left popliteal fossa (Fig. 2).

The tick appeared to be swollen and was constantly biting the patient; it was subsequently removed by using fine-tipped tweezers. Hence, the patient was suspected of having SFTS. A viral genome detection test was performed via reverse transcription–polymerase chain reaction (RT–PCR; Yoshikawa et al. 2014) using total RNA extracted from a peripheral blood sample, a pharyngeal swab, and a urine specimen obtained on the day after admission. Three days after the initial test (hospital day 5), the test was repeated for whole blood and urine specimens. In the pharyngeal swab, the viral gene copy number (S segment) was 10^5.08^/mL on hospital day 2 (6 days after disease onset). In the whole blood, the viral gene copy number was 10^4.43^/mL on hospital day 2, but had decreased to 10^2.92^/mL on hospital day 5.

**Fig. 3:**
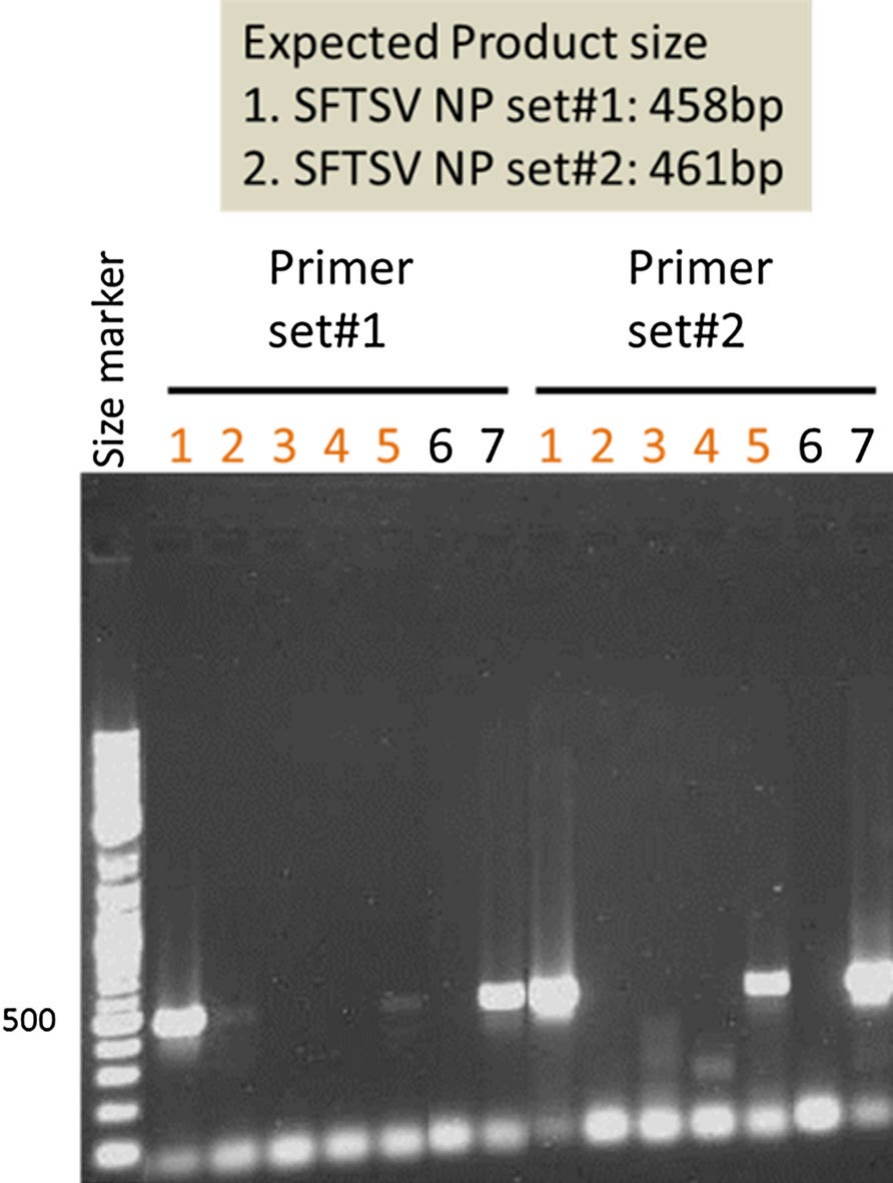
SFTS viral genome analysis by RT-PCR for case 1. Primer sets were prepared for the Japanese congenital SFTS virus. To increase detection sensitivity, two primer sets for different target areas were used. *Lanes 1, 3, and 5* represent the samples (serum, urine, and pharyngeal swab, respectively) taken on the day after admission. *Lanes 2 and 4* represent samples (serum and urine respectively) taken 3 days after the initial test. *Lanes 6 and 7* contain a negative and positive control, respectively

Prior to the first viral genome detection test, the patient began treatment with 60 mg (1 mg/kg) prednisolone orally for hemophagocytic syndrome and 100 mg minocycline twice a day intravenously for a suspected Rickettsial infection. Four days after treatment initiation, her symptoms improved, and 5 days after treatment initiation, the whole blood cell count, liver enzyme levels (AST, ALT, and LDH), and ferritin values returned to normal. Ten days after admission, the tick was identified as *Haemaphysalis longicornis*, and a definitive diagnosis of SFTS was made by conventional viral genome amplification analysis (Fig. 3; Yoshikawa et al. 2014). The test results showed that the viral genome was amplified in the serum and swab specimens collected on hospital day 2. In the second test after treatment, the viral genome could not be amplified from the urine specimen collected on hospital day 5. By this time, prednisolone and minocycline treatment had been terminated because hemophagocytic syndrome was cured and the result of the Rickettsial infection test was negative. Subsequently, the results of a whole blood test were normal, and the patient’s general status was significantly improved.

Following the treatment, the patient was discharged from the hospital, returned to her usual lifestyle, and remains symptom-free in the 2 years post-treatment.

### Case 2

An 82-year-old woman visited a primary care clinic owing to diarrhea and general fatigue.

She had hypertension but received no treatment at this time in the clinic. Because she was senseless and listless, she was referred to a community hospital on the following day. The results of a complete blood count were as follows: WBC count, 1060/µL (lymphocyte count, 230/µL); RBC count, 348 × 10^4^/µL; Hb level, 10.3 g/dL; and platelet count, 5.1 × 10^4^/µL. On the same day, she was referred to the Division of General Medicine at the hospital for further evaluation and treatment. When she presented at the hospital, her consciousness level was defined as groggy [GCS, 11 points (eye opening, 4; verbal response, 2; and motor response, 5)]. She had a body temperature of 37.7 °C, a blood pressure of 125/55 mmHg, and a pulse rate of 93 beats/min (regular). There were no remarkable findings on her physical examination. The laboratory findings were as follows: AST level, 274 IU/L; ALT level, 61 IU/L; LDH level, 935 IU/L; CPK level, 1485 IU/L; BUN level, 44.1 mg/dL; Cr level, 1.52 mg/dL; fibrinogen level, 126.2 mg/dL; fasting triglyceride level, 110 mg/dL; sIL-2R level, 3030 U/mL; and ferritin level, 3383 ng/mL. There were no remarkable findings on chest radiography or head computed tomography.

**Fig. 4:**
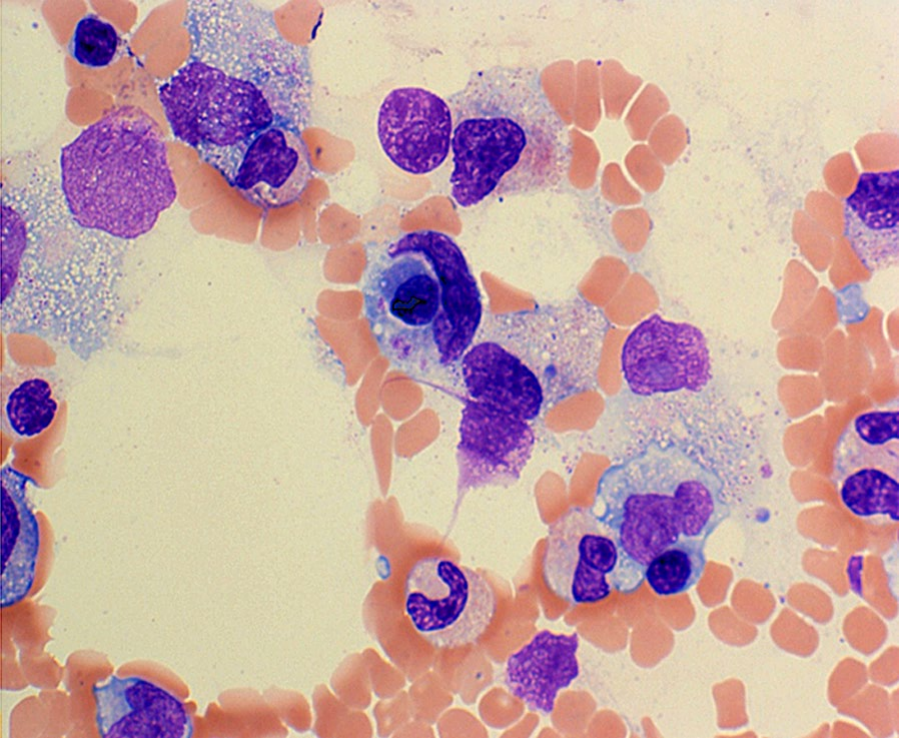
Bone marrow smear findings for case 2. Platelets are ingested by a macrophage (May-Giemsa staining, ×400 magnification)

Abdominal computed tomography showed bilateral lymph node swelling in the para-aortic and inguinal regions. However, there was no splenomegaly. On hospital day 3, 4 days after disease onset, the patient had a high fever and severe diarrhea. A bone marrow biopsy and a smear test were performed, revealing that platelet cells were being ingested by macrophages (Fig. 4). The patient met six of the eight HLH2004 diagnostic criteria, and hence, hemophagocytic syndrome was diagnosed. Although a workup was performed to determine the cause of this syndrome, no previously described etiologic agents or diseases were detected. Methylprednisolone at 1000 mg/day was administered for three consecutive days, and cefepime at 2 g was administered intravenously thrice daily. However, the patient’s general condition worsened, and the results of the laboratory tests were exacerbated. On hospital day 6, the laboratory results were as follows: WBC count, 5900/µL; RBC count, 332/µL; platelet count, 1.9 × 10^4^/µL; LDH level, 1583 IU/L; BUN level, 114.8 mg/dL; and Cr level, 4.58 mg/dL. The patient’s condition deteriorated progressively, and she died on hospital day 8. The patient’s family was informed, and an autopsy was performed.

**Fig. 5:**
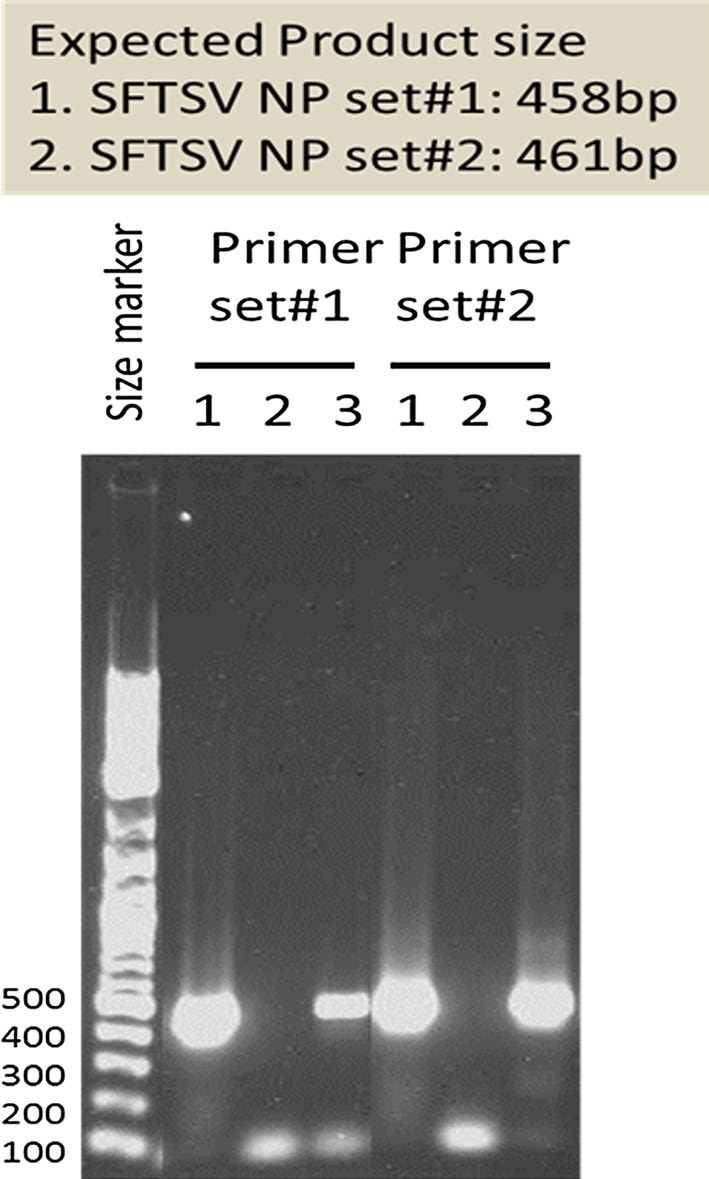
SFTS viral genome analysis by RT-PCR for case 2. Primer sets were prepared for the Japanese congenital SFTS virus. To increase detection sensitivity, two primer sets for different target areas were used. *Lane 1* represents a frozen serum specimen, *lane 2* contains a negative control, and *lane 3* contains a positive control

Histological analysis revealed hemophagocytic lesions in multiple organs. There was no evidence of malignancy. As the patient had died, a frozen peripheral blood serum collected on hospital day 2 (3 days after disease onset) was tested for SFTS viral genome amplification via RT-PCR, and amplification was observed (Fig. 5). The patient was retrospectively diagnosed with SFTS. The viral gene copy number was 1 × 10^7.09^. There was no evidence of tick bites in the patient.

## Discussion and evaluation

Herein, we describe two patients who were diagnosed with SFTS; one patient survived (patient 1) and the other died (patient 2). There were two main differences in the clinical course of these patients. First, their consciousness levels in the acute phase (within the first week of onset) differed: a GCS of 14 points in the patient who survived and a GCS of 11 points in the patient who died. Although both patients were immediately treated with steroids at our hospital, their response to therapy was different. Possible explanations include the following: (1) patient 2 was admitted to our hospital later than patient 1 relative to the onset of symptoms, and thus, her treatment was delayed, (2) patient 2 had higher levels of liver enzymes and CPK during the course of treatment than did patient 1, and (3) patient 2 was older than patient 1. In addition, central nerve system manifestations, particularly lethargy and coma, are correlated with mortality (Gai et al. 2012a).

In the patient who survived, the consciousness level normalized after steroid therapy.

Laboratory parameters also normalized within 12 days after symptom onset. In the patient who died, the consciousness level deteriorated with time, and the laboratory parameters worsened, particularly the platelet counts, liver enzyme levels, CPK levels, and renal function test measurements. These differences in the clinical course may be related to the viral load (Yoshikawa et al. 2014). The viral gene copy number was low and decreased immediately in the patient who survived but not in the patient who died. Viral load is related to morbidity (Yoshikawa et al. 2014; Sun et al. 2012; Gai et al. 2012b) and may therefore correlate with consciousness level, which could therefore be used to predict death in SFTS patients. In contrast to consciousness level, which is quickly and easily assessed, determination of viral gene copy number requires several days, and laboratory tests for viral load can only be performed at a limited number of inspection institutes (for example, the National Institute of Infectious Disease). In addition, a recent study showed that increased AST, LDH and CPK levels, decreased lymphocyte percentages, and older ages were significantly associated with the death of patients with SFTS (Sun et al. 2012; Zhang et al. 2012b; Deng et al. 2013; Cui et al. 2013).

The two patients described in this report were similar in that both had hemophagocytic syndrome, which has been shown to correlate with cytokine levels and the severity of SFTS (Sun et al. 2012). Steroids are traditionally administered for treatment of hemophagocytic syndrome in many cases, but the efficacy is unclear. Usually, hemophagocytic syndrome occurs following other conditions, most notably lymphoma and Epstein-Barr virus infection. In such cases, a patient receives a specific therapy: chemotherapy for malignant lymphoma and an etoposide-containing regimen for Epstein-Barr virus infection associated with severe hemophagocytic syndrome (Jordan et al. 2011). These causes did not apply to the patients in our report, and therefore, these therapies were not chosen.

The specific treatment for SFTS has not been established. A recent study showed that administration of ribavirin or tetracycline did not prolong the survival of SFTS patients (Liu et al. 2013). Combined usage of type-I/II IFN and ribavirin drastically reduced SFTS virus infection in vitro and therefore may be useful in the treatment of SFTS (Shimojima et al. 2015). When a case of SFTS is diagnosed, supportive care should be provided to improve the general condition of the patient.

## Conclusions

We encountered two cases of SFTS; one patient survived but the other died. Differences between the clinical and biological parameters of the two patients included age, time of treatment relative to symptom onset, level of consciousness, LDH level, and CPK level. We suggest that viral load is an important predictor of morbidity and is associated with the level of consciousness and the serum LDH level. As shown in our previous report, these parameters correlated with SFTS prognosis, and they may therefore be useful for predicting death in SFTS patients.

## Abbreviations

ALT: alanine aminotransferase
AST: aspartate aminotransferase
BUN: blood urea nitrogen
CPK: creatinine phosphokinase
Cr: serum creatinine
GCS: Glasgow Coma Scale
Hb: hemoglobin
IFN: interferon
LDH: lactate dehydrogenase
RBC: red blood cell
SFTS: severe fever with thrombocytopenia syndrome
sIL-2R: soluble interleukin-2 receptor
WBC: white blood cell
